# Electro-acupuncture on Vascular Parkinsonism with multiple sleep disorders: A Case Report

**DOI:** 10.3389/fneur.2022.1057095

**Published:** 2022-12-19

**Authors:** Mingyue Yan, Jingqi Fan, Yingjia Li, Xin Liu, Zhengmiao Yu, Lixing Zhuang

**Affiliations:** ^1^Clinical Medical College of Acupuncture-Moxibustion and Rehabilitation, Guangzhou University of Chinese Medicine, Guangzhou, China; ^2^Acupuncture Clinic, Rehabilitation Center, The First Affiliated Hospital of Guangzhou University of Chinese Medicine, Guangzhou, China

**Keywords:** Electro-acupuncture, Vascular Parkinsonism, sleep disorder, case report, polysomnography

## Abstract

Vascular Parkinsonism (VP) is a kind of rare secondary Parkinsonism caused by vascular lesions. Patients with VP experience not only movement disorders but also sleep disorders. But treatment options are limited and often associated with undesirable adverse effects. Electro-acupuncture (EA) is a safe, rapid work, easy operation, and convenient complementary replacement therapy. We report a case of a 51-year-old man who presented with VP and multiple sleep disorders. Based on clinical evaluation and nocturnal hospital-based polysomnography (PSG), the patient had severe PLMD (PSG showed severe periodic leg movements), excessive daytime sleepiness (EDS, the score of the ESS is 16), and probable rapid eye movement sleep disorder (RBD). Parkinson's disease sleep scale (PDSS) score, Pittsburgh sleep quality index (PSQI), and periodic leg movements index were 93, 11, and 135.2, respectively. After 8 weeks of EA treatment, the patient reported that the symptoms of subjective and objective sleep disturbance were significantly alleviated without any discomfort. This case report may provide a new alternative and complementary therapy for VP patients with sleep disturbance but more definitive and robust evidence is needed to support its efficacy.

## Introduction

Vascular Parkinsonism (VP) is a kind of rare secondary Parkinsonism caused by vascular lesions and accounts for 3–5% of all patients with Parkinsonism (1). Clinical presentation of classical VP has been described as lower body Parkinsonism, characterized by impaired gait (1). Patients with VP show short stride length, gait opening failure, and poor postural response to maintain balance. These symptoms remain stable or gradually deteriorate. Therefore, in most cases, the onset is insidious (2). At the same time, patients with VP experience non-motor symptoms but these have been relatively neglected in the neurological literature until recent years. A PRIAMO study found a 70.7% prevalence of sleep disturbance in VP groups (3). Nocturia is the most frequent and troublesome nocturnal symptom (3). Different sleep disturbances can be observed in patients with VP. Subjective sleep disturbances include difficulty in falling asleep, waking early, sleep fragmentation, and RLS. Objective sleep disturbances include periodic limb movements (PLMS), REM sleep behavior disorder (RBD), obstructive sleep apnea (OSA), and excessive daytime sleepiness (EDS) (4).

The diagnosis and treatment of VP have posed a challenge to neurologists. When patients are thought to be afflicted with both sleep disorders and movement disorders, the situation becomes more complicated. Furthermore, due to the wide range of sites of onset, patients may have a combination of several unusual sleep disturbances at the same time. Due to the lack of prospective research on the coexistence of VP with sleep disorders such as insomnia, PLMS, and RLS, the most commonly applied therapy is the same as in the general population. Behavioral, psychological, and pharmaceutical treatments are available (5), but their duration of effectiveness is constrained. There is a high risk of rebound or other side effects.

Electro-acupuncture (EA) is a valuable non-pharmacological therapy in the management of neurological diseases. It functions when acupuncture needles are linked to a modest amount of low-frequency pulse current *via* an EA machine. It has the advantages of safety, rapid work, easy operation, and convenience; hence, EA can be easily promoted to patients. Moreover, the effectiveness of EA in treating sleep disorders along with insomnia after stroke has been confirmed in some systematic reviews and overviews (6–8). Based on preliminary research, we speculated EA can improve sleep disorders in patients with VP. As a result, we report a VP patient with several sleep disorders whose subjective and objective sleep disturbances were relieved after 8 weeks of EA therapy. This study was approved by the Ethics committee of the First Affiliated Hospital of Guangzhou University of Chinese Medicine.

## Case description

### Clinical history

The patient, a 51-year-old man with a 2-year history of Parkinsonism, presented with motor retardation, sleep disturbance, and slurred speech as his main complaints. The patient cannot start and rise from the seat independently, when he began walking, he had a slow and insecure gait. Posture is unstable, and postural responses to maintain balance are poor (1). He also had a mild rest tremor in both upper limbs, mostly on the right. At the same time, slurred speech, frequent drooling, and difficulty in opening his mouth also negatively affected his common life. Besides, the patient's wife told us the patient kicked and shouted at night, snored, urinated 3–4 times per night, and felt fatigued, sleepy, and drowsy during the daytime. We observed the patient frequently dozed off during conservation.

The patient's symptoms began at 47 with a slight tremor in his right hand, which was ignored initially because it did not significantly disrupt his life. Until 2020, the symptoms affected speech and movement, making it harder to carry out daily tasks. He was diagnosed with Parkinsonism and prescribed Levodopa and Benserazide Hydrochloride Tablets 0.125 g [State Drug Certificate H10930198] twice daily and Pramipexole Dihydrochloride Tablets 0.5 mg [H20193413] three times a day. According to the cranial MR+MRA at that time: (1) the Bilateral posterior cerebral artery P2 segment was significantly narrowed. (2) Multiple ischemic foci in both frontoparietal lobes. The Cranial PET-CT revealed bilateral striatal dopamine metabolism was decreased, which was obvious on the left side; bilateral basal ganglia glucose metabolism was decreased, which was obvious on the left side. The aforementioned imaging abnormalities were consistent with PDS metabolic changes and did not support Parkinson's disease (PD). The relevant pictures are in the attachment.

## Physical examination and polysomnography

The patient had clear consciousness, slurred speech, free eye movements, no nystagmus, less facial expression, difficulty in opening mouth and tongue extension, forward gait, normal muscle strength of all four limbs, cogwheel-like increase in muscle tone of both upper limbs, active biceps tendon reflex, weak knee, and Achilles tendon reflexes, stiff neck and limb joints, positive Babinski's sign on the left side, and positive jaw reflex. His PDSS score was 93, PSQI score was 11, and Epworth Sleepiness Scale (ESS) score was 16.

**Figure 1:**
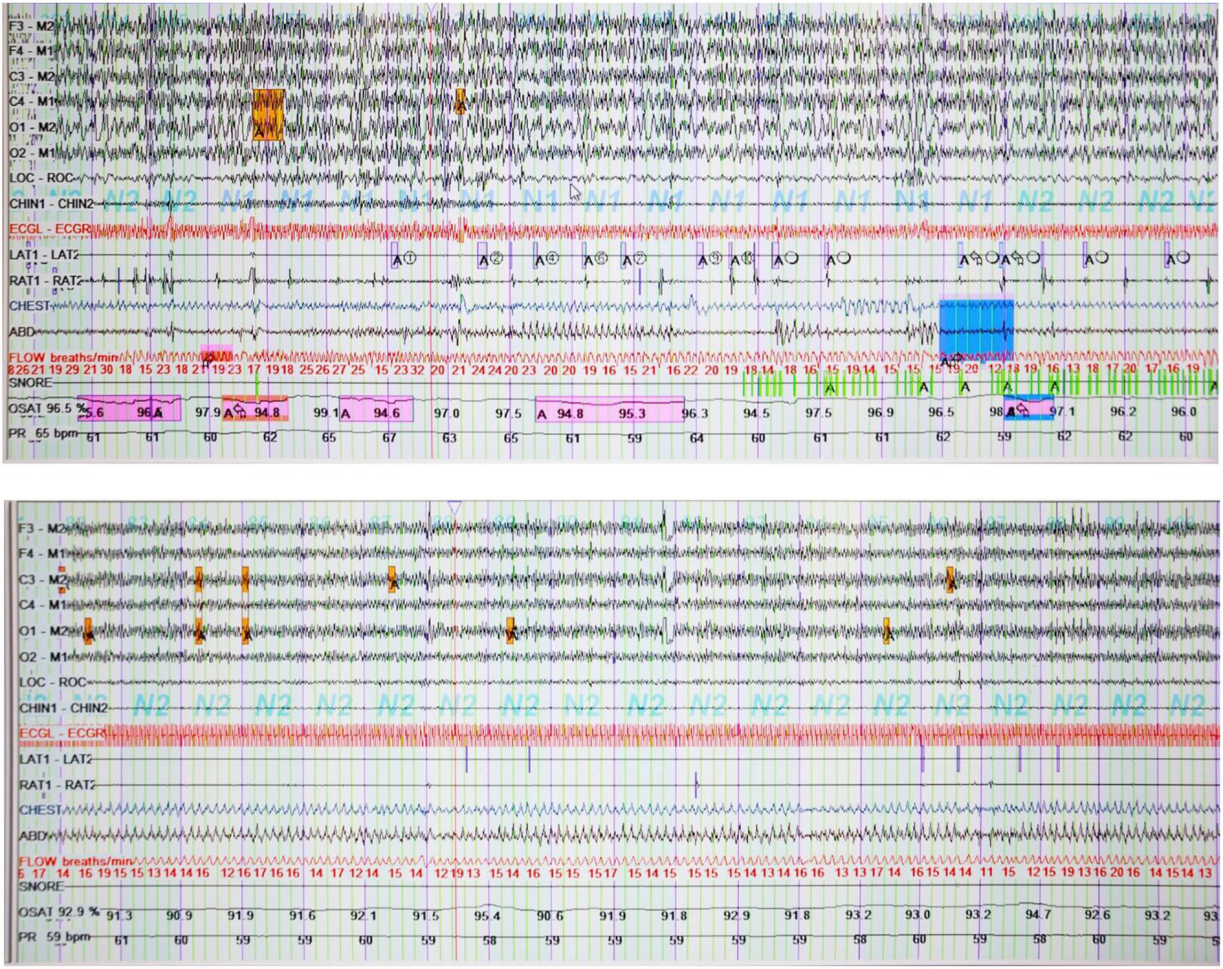
Polysomnography before and after treatment. AT, electromyography of anterior tibialis left (L) and right (R) muscles; LAT1-LAT2 shows repetitive stereotypical movements in the left leg before EA treatment. It was recorded 792 times during sleep; OSAT, oxyhemoglobin saturation.

The polysomnography (PSG) suggested that the patient had reduced sleep efficiency and prolonged Wake-time After Sleep Onset (WASO) time. The minimum oxygen saturation of 83.3%, which is regarded as moderate hypoxemia, was recorded during sleep. The following sleep structures were recorded: N1 35.4, N2 57.2, N3 1.0, and REM 6.4%. Periodic leg movements were recorded 792 times during sleep with an index of 135.2, which was considered severe periodic leg movements (Figure 1).

## Acupuncture treatment

**Figure 2:**
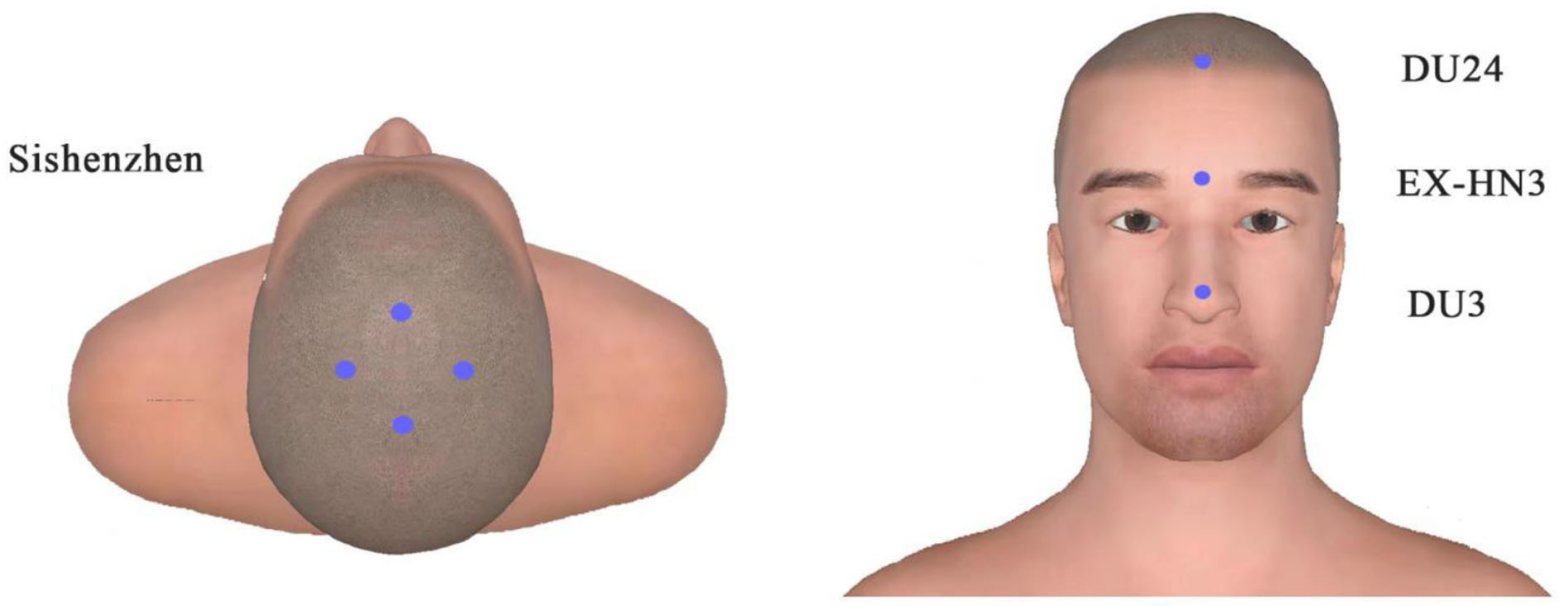
Locations of head acupoints.

**Figure 3:**
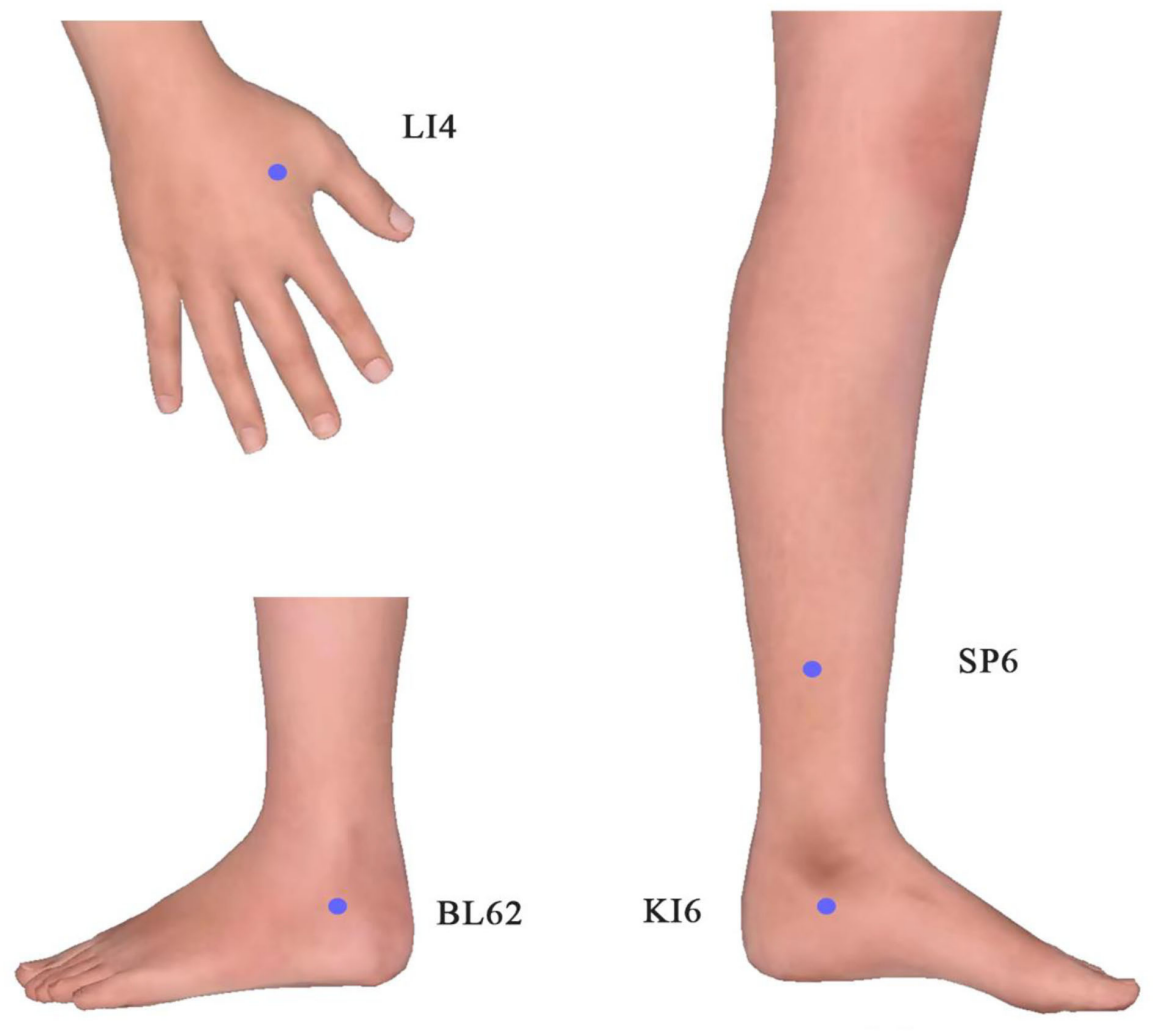
Locations of limb acupoints (both sides).

We performed EA treatment for 8 weeks. And the following 15 acupoints were selected: Sishenzhen (9), Shenting (DU24), Yintang (EX-HN3), Suliao (DU25) as the head acupoints, and Hegu (LI4), Shenmai (BL62), Zhaohai (KI6), Sanyinjiao (SP6). Figures 2, 3 display these acupoints' locations. After skin disinfection, the acupuncturist placed stainless steel needles at the appropriate angle under the patient's skin. The acupuncturist employed a constant direct current for 30 min at a setting of 1–2 mA with a frequency of 20 Hz until the patient felt a sense of deqi (i.e., soreness, numbness, warmth, heaviness, or swelling around the acupuncture points). The EA treatment lasted 30 min per session and was administered four times a week for 8 weeks. The therapy was performed by an acupuncturist registered in China with 5 years of practice experience. The patient maintained the original doses of his anti-Parkinson medications. No adverse events were observed during the treatment.

After treatment, the patient's wife reported a reduction in the severity and frequency of snoring and limb movements at night. Repeated sleep PSG revealed: (1) Sleep efficiency was reduced, latency was normal, and WASO time was prolonged. (2) Sleep structure: N1 stage 58.8, N2 stage 32.1, N3 stage 0, and REM stage 9.2%. (3) AHI was 3.0, which was within the normal range. Oxygen saturation was over 88 of 99.9% of the time, and the lowest oxygen saturation was 86.7%, which is mild hypoxemia. (4) A total of 405 periodic leg movements were recorded while sleeping, with an index of 64.5. Due to the distance, the patient returned home for recuperation. There was no significant change in the condition after a month of telephone follow-up.

## Discussion

Vascular Parkinsonism is used to describe a clinical phenotype characterized primarily by gait impairment and associated with vascular pathogenesis that primarily affected subcortical white matter (10). The patient's condition can be characterized by abnormal gait (difficulty in rising, reduced stride length, forward gait), extrapyramidal symptoms (tremor, increased muscle tone), pseudobulbar palsy (poor speech, difficulty in opening mouth and extending tongue), poor levodopa reaction, and multiple ischemic foci. Besides, the patient had severe PLMD (PSG examination showed severe periodic leg movements, with an index of 135.2), excessive daytime sleepiness (EDS), and possible rapid eye movement sleep disorder (RBD). Based on the patient's medical history, symptoms, signs, and auxiliary examinations, we concluded that the patient had VP with a combination of multiple sleep disorders. Multiple sleep disturbances can raise the possibility of poor living quality, depressed mood, decreased cognition, and even falls, all of which will negatively impact the disease's prognosis and lay much more burden on the family and society (11). Altogether, sleep disorders in patients with VP should be identified and treated as soon as possible.

For VP patients with sleep disorders, current pharmaceutical treatment options are quite limited and often associated with undesirable adverse effects. Benzodiazepines are commonly used to treat insomnia and may cause morning sedation, confusion, and imbalance in the movement disorder population. Dopamine agonists (12), Fe supplementation, clonazepam, and opioids are the most commonly used drugs for the treatment of RLS/PLMS but may lead to excessive daytime sleepiness, predisposing patients to falls overnight, or causing confusion. Non-pharmacological treatment approaches can be the main approach for EDS, especially for its milder forms (13).

Electro-acupuncture is a characteristic traditional Chinese non-pharmacological method. It not only can improve sleep architecture (14) and prolong deep sleep time in patients with insomnia (15) but also can improve motor symptoms in patients with movement disorders (16). Polysomnography (PSG) is considered the gold standard for the diagnosis of non-rapid eye movement (NREM) parasomnias (17), particularly valuable for individuals with complex medical histories. In this case, EA was selected as the treatment, and PSG was used to monitor the patient's sleep. The acupuncturist selected Sishenzhen, Shenting (DU24), Yintang (EX-HN3), and Suliao (DU25) as the head acupoints and Hegu (LI4), Shenmai (BL62), Zhaohai (KI6), and Sanyinjiao (SP6) as the distal points of the limbs. The mechanism is still unclear. Studies have suggested that promoting neurogenesis, alleviating neuroinflammation, inhibiting neuronal cell apoptosis, and regulating oxidative stress may all be involved in how acupuncture functions in nervous system disease (18). To be specific, EA at the head may alleviate sleep-related functional impairment caused by nerve function damage. EA at the body acupoints may enhance blood flow, release neuromediators that regulate physical functions, calm the body to induce sleepiness, and improve the quality of sleep. A cluster randomized crossover pilot study showed pressing leg acupoints decreased aberrant leg activity and nighttime activity in patients with RLS (19). A study demonstrated that EA activated the signal of BDNF/TrkB/Erk to promote the survival and synaptic plasticity of hippocampal neurons, which alleviates spatial memory impairment induced by sleep deprivation (20).

The scores of the severity of the patient's nocturnal sleep and leg movement index showed a severe level at first. After EA treatment, subjective sleep improved a lot. PDSS scores increased by 8 points and PSQI scores decreased by 2 points, with changes mainly in difficulty falling asleep and energy recovery. Besides, long deep sleep, lower PLM index (70.7↓), lower AHI index (1.3↓), lower ODI score (9.1↓), and higher oxygen saturation with PSG were all indicators of improvement of objective sleep (Specific results in Table 1). But there was no significant change in daytime sleepiness and urination. The reason could be no specific acupoints for these symptoms. Besides, the second PSG examination clearly shows a longer awakening time, we infer that patients with VP typically experience unstable and worsening sleep as the disease progresses. After the 1-month follow-up, the situation of leg movements and subjective sleep remained stable, but the nocturnal urination problem was similar to the previous situation, and the patient continued to doze off sometimes during the day.

**Table 1:** The parameter changes before and after EA treatment.

		**Before**	**After**
Polysomnography	Time in bed(min)	432.6	573.5
	Total sleep time (min)	351.5	377
	Sleep efficiency(%)	81.3	65.7
	Sleep-onset latency (min)	6.7	6.7
	Wake after sleep onset (min)	81.5	197
	N1 sleep (min)	124.5	221.5
	N2 sleep (min)	201	121
	N3 sleep (min)	3.5	/
	REM sleep (min)	22.5	34.5
	AHI score (events/h)	4.3	3.0
	ODI score (events/h)	14.0	4.9
	PLM Index (events/h)	135.2	64.5
Scales	PDSS	93	101
	ESS	16	16
	PSQI	11	9
	HMAD	9	8
	HAMA	5	5

REM sleep, rapid-eye-movement sleep; AHI, apnea-hypopnea index; ODI, oxygen desaturation index; PDSS, score ranging from 0 to 150, with higher scores indicating more serious sleep disturbance; ESS, score ranging from 0 to 24, with higher scores indicating more serious sleepiness; PSQI, 0–5 stands for good sleep, and higher scores indicating worse sleep; HAMD, 0–7 stands for normal,7–16 stands for probable depression,17–24 stands for depression, with higher scores indicating more serious depression; HAMA, 0–7 stands for normal,7–13 stands for probable anxiety,14–56 stands for anxiety, with higher scores indicating more serious anxiety.

## Conclusion

We presented a VP patient with multiple sleep disorders who responded to EA therapy positively. Meanwhile, no adverse effects were observed. The study confirms that EA is beneficial to subjective and objective sleep in a patient with VP. The application of PSG enables our diagnosis and efficacy assessment more subjective. We are unaware of any other articles reporting on the relief of sleep disorders by acupuncture in patients with VP.

Although the significant results in our study, the PDSS and PSQI scores, and PLM index still remained above the clinical cut-off level, indicating that the patient needs a long-term EA treatment to maintain the effects.

## Data availability statement

The original contributions presented in the study are included in the article/Supplementary material, further inquiries can be directed to the corresponding author.

## Ethics statement

The studies involving human participants were reviewed and approved by the Ethics committee of the First Affiliated Hospital of Guangzhou University of Chinese Medicine. The patients/participants provided their written informed consent to participate in this study. Written informed consent was obtained from the individual(s) for the publication of any potentially identifiable images or data included in this article.

## Author contributions

MY conceived the idea and prepared the manuscript. JF conceptualized the research. XL collected and analyzed the data. YL assisted in clinical treatment. ZY conducted the PSG and assisted in clinical diagnosis. LZ revised the manuscript. All authors contributed to the article and approved the submitted version.
