# Supplementary material for: Electro-acupuncture on Vascular Parkinsonism with multiple sleep disorders: A Case Report
Source: Front Neurol. 2022 Dec 19;13:1057095. doi: 10.3389/fneur.2022.1057095 (PMC9806161; doi:10.3389/fneur.2022.1057095)
Supplement: Supplementary file 1 [file Data_Sheet_1.PDF]

广州中医药大学第一附属医院
多导睡眠监测报告

患者姓名:	伦进良	检查日期:	8/11/2022
出生日期:	9/11/1971	检查类型:	Master
年龄:	50 岁	脑电图号:	20220811s
性别:	男	住院号:	661592
科室:	脑病科	床号:	40

睡眠整体情况浏览

第一次关灯时间:	09:09:09 PM
最后一次开灯时间:	06:42:38 AM
总的卧床时间:	573.5
总的睡眠时间:	377.0
睡眠效率:	65.7%
睡眠周期时间:	560.0
睡眠维持效率:	67.3%
睡眠潜伏期:	6.7
REM 潜伏期:	15.0

	COUNT	INDEX
清醒情况:	62	9.9
觉醒反应:	266	42.3
所有 通气不足:	46	7.3
肢体动作:	459	73.1
打鼾:	-	-
血氧饱和度:	31	4.9
最低血氧饱和度:	86.7%	

睡眠结构分析

睡眠分期	时间 (mins)	% 百分比
觉醒	197.0	
1 期睡眠	221.5	58.8%
2 期睡眠	121.0	32.1%
3 期睡眠	-	0.0%
REM 睡眠	34.5	9.2%

% of Time in Bed

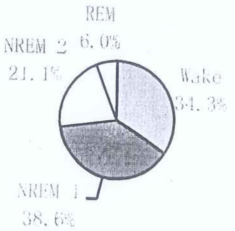

觉醒情况分析

	NREM	REM	Total Sleep Time
呼吸暂停 & 通气不足导致觉醒	4	-	4
肢体运动导致觉醒	10	2	12
孤立肢体运动导致觉醒	1	-	1
自发觉醒	84	14	98
合计	98	17	115
觉醒指数	17.2	29.6	18.3

呼吸情况分析

	By Sleep Stage			TOTAL
	NREM	REM	Wake	
时间 (min)	342.5	34.5	0.0	377.0
呼吸暂停合计	2	-	-	3
总的呼吸暂停指数	0.4	-	-	0.5
**Hypopneas w/Arousals**	4	-	-	4
**Hypopneas w/≥3% Ds** (incl. Ds only)	11	1	-	12
**Total Hypopneas (≥3%)** (Hyp w/Ar + Hyp w/≥3% Ds)	15	1	-	16
**Tot. Hypopnea Index (≥3%)** (Hyp w/Ar + Hyp w/≥3% Ds)	2.6	1.7	-	2.5
**Hypopneas w/≥4% Ds**	11	1	-	12
**Total Hypopneas (≥4%)** (Hyp w/Ar + Hyp w/≥4% Ds)	15	1	-	16
**Tot. Hypopnea Index (≥4%)** (Hyp w/Ar + Hyp w/≥4% Ds)	2.6	1.7	-	2.5
**All Apneas & Hypopneas** All Apn + Hyp w/Arousal + Hyp with Desat Only	17	1	1	19
**AHI** (All Apn + Hyp w/Ar + Hyp. w/Ds)	3.0	1.7	0.3	3.0
**Apneas & Hyp. (≥3%)** All Apn + Hyp w/Arousals + Hyp w/≥3% Desat	17	1	1	19
呼吸暂停低通气指数 AHI **AHI (≥3% Criteria)** Index of All Apn + Hyp w/Ar + Hyp. w/≥3% Desat	3.0	1.7	0.3	3.0
呼吸暂停及低通气 **Apneas & Hyp. (≥4%)** All Apn + Hyp w/Arousals + Hyp w/≥4% Desat	17	1	1	19
呼吸暂停低通气指数 AHI **(≥4% Criteria)** Index of All Apn + Hyp w/Arousals + Hyp w/≥4% Desat	3.0	1.7	0.3	3.0

呼吸事件小结

	呼吸暂停		呼吸不足	
	NREM	REM	NREM	REM
平均 (s)	10.6	-	20.1	16.7
最大值(s)	13.2	-	41.0	30.0

血氧饱和度情况小结

	WAKE	NREM	REM
平均血氧饱和度 (%)	95.3%	94.7%	94.4%
最低血氧饱和度 (%)	86.6%	86.7%	88.7%
最高血氧饱和度 (%)	99.6%	99.0%	97.5%

血氧饱和度分布

范围(%)	时间(min)	时间占比(%)
90.0 - 100.0	571.7	99.7%
80.0 - 90.0	1.7	0.3%
70.0 - 80.0	-	-
60.0 - 70.0	-	-
50.0 - 60.0	-	-
0.0 - 50.0	-	-

血氧饱和度低于 **88%**的时间

范围(%)	时间 (min)	时间占比 (%)
0.0 - 88.0	0.5	0.1%

血氧饱和度下降	31
血氧下降过程中最低血氧饱和度	86.7%

肢体运动情况小结

	COUNT	INDEX
孤立肢体运动	54	8.6
周期性肢体运动 (PLMs)	405	64.5
肢体运动合计	459	73.1

脉搏情况小结

	WAKE	NREM	REM	Sleep	TOTAL
平均脉搏 (BPM)	61.7	62.8	61.1	62.6	62.3
最低脉搏 (BPM)	54.8	35.0	52.9	35.0	35.0
最快脉搏 (BPM)	78.6	81.7	71.1	81.7	81.7

脉搏分布

脉搏范围(bpm)	时间 (min)	时间占比 (%)
0.0 - 40.0	0.2	0.0%
40.0 - 60.0	179.3	31.3%
60.0 - 80.0	393.9	68.7%
80.0 - 100.0	0.0	0.0%
100.0 - 120.0	-	-
120.0 - 140.0	-	-
140.0 - 200.0	-	-

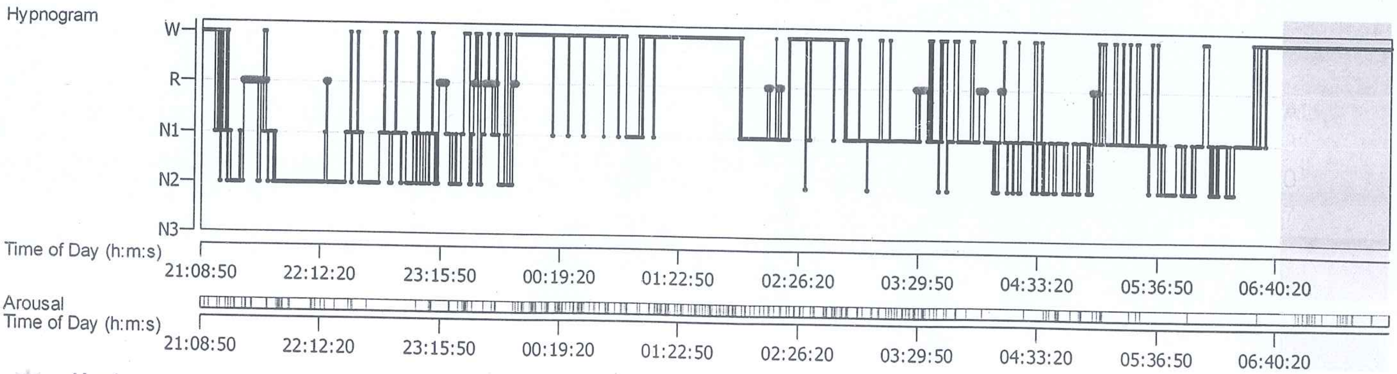

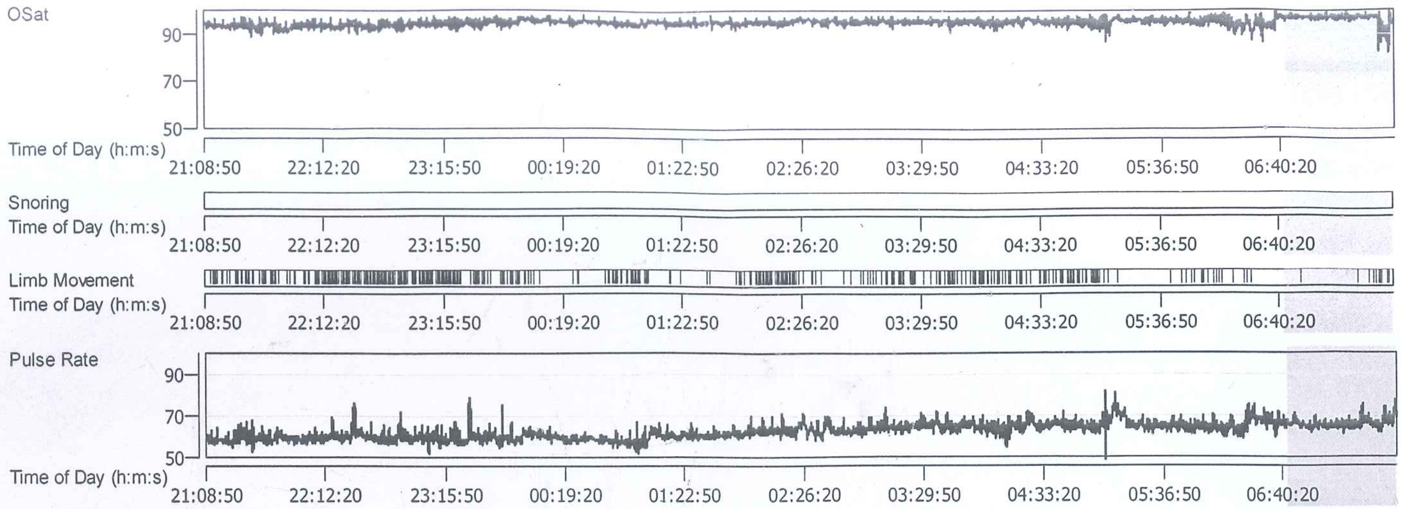

小　结

1.睡眠效率降低，潜伏期正常，WASO 时间延长。

2.睡眠结构：N1 期 58.8%，N2 期 32.1%，N3 期 0%，REM 期 9.2%。REM 潜伏期 15min。

3.睡眠期间记录到呼吸暂停-低通气状态 19 次，AHI 为 3.0，属于正常范围；血氧饱和度大于 88%的时间占 99.9%，最低血氧饱和度 86.7%，属于轻度低氧血症。

4.睡眠期间记录到周期性腿动 405 次，指数为 64.5，考虑属于重度周期性腿动。

报告医师：于征淼
2022 年 8 月 12 日
