# Supplementary material for: Electro-acupuncture on Vascular Parkinsonism with multiple sleep disorders: A Case Report
Source: Front Neurol. 2022 Dec 19;13:1057095. doi: 10.3389/fneur.2022.1057095 (PMC9806161; doi:10.3389/fneur.2022.1057095)
Supplement: Supplementary file 2 [file Data_Sheet_2.PDF]

CARE
case report guidelines

CARE Checklist of information to include when writing a case report

(cc) BY-NC-ND

Topic	Item	Checklist item description	Reported on Line
**Title**	1	The diagnosis or intervention of primary focus followed by the words "case report"	4-5
**Key Words**	2	2 to 5 key words that identify diagnoses or interventions in this case report, including "case report"	12-13
**Abstract (no references)**	3a	Introduction: What is unique about this case and what does it add to the scientific literature?	14-27
	3b	Main symptoms and/or important clinical findings	20-24
	3c	The main diagnoses, therapeutic interventions, and outcomes	20-22, 24-25
	3d	Conclusion—What is the main "take-away" lesson(s) from this case?	26-27
**Introduction**	4	One or two paragraphs summarizing why this case is unique (**may include references**)	29-58
**Patient Information**	5a	De-identified patient specific information.	61-62
	5b	Primary concerns and symptoms of the patient.	63-68
	5c	Medical, family, and psycho-social history including relevant genetic information	69-79
	5d	Relevant past interventions with outcomes	71-74
**Clinical Findings**	6	Describe significant physical examination (PE) and important clinical findings.	81-86
**Timeline**	7	Historical and current information from this episode of care organized as a timeline	69-79
**Diagnostic Assessment**	8a	Diagnostic testing (such as PE, laboratory testing, imaging, surveys).	87-92
	8b	Diagnostic challenges (such as access to testing, financial, or cultural)	41-44
	8c	Diagnosis (including other diagnoses considered)	116-123
	8d	Prognosis (such as staging in oncology) where applicable	123-125
**Therapeutic Intervention**	9a	Types of therapeutic intervention (such as pharmacologic, surgical, preventive, self-care)	94-96
	9b	Administration of therapeutic intervention (such as dosage, strength, duration)	97-102
	9c	Changes in therapeutic intervention (with rationale)	103-104
**Follow-up and Outcomes**	10a	Clinician and patient-assessed outcomes (if available)	105-106
	10b	Important follow-up diagnostic and other test results	111-112
	10c	Intervention adherence and tolerability (How was this assessed?)	102-106
	10d	Adverse and unanticipated events	104
**Discussion**	11a	A scientific discussion of the strengths AND limitations associated with this case report	134-138
	11b	Discussion of the relevant medical literature **with references**.	114-158
	11c	The scientific rationale for any conclusions (including assessment of possible causes)	139-148
	11d	The primary "take-away" lessons of this case report (without references) in a one paragraph conclusion	160-167
**Patient Perspective**	12	The patient should share their perspective in one to two paragraphs on the treatment(s) they received	105-106, 111-112
**Informed Consent**	13	Did the patient give informed consent? Please provide if requested	Yes ☑ No ☐
