# Supplementary figures and images for: Electro-acupuncture on Vascular Parkinsonism with multiple sleep disorders: A Case Report
Source: Front Neurol. 2022 Dec 19;13:1057095. doi: 10.3389/fneur.2022.1057095 (PMC9806161; doi:10.3389/fneur.2022.1057095)

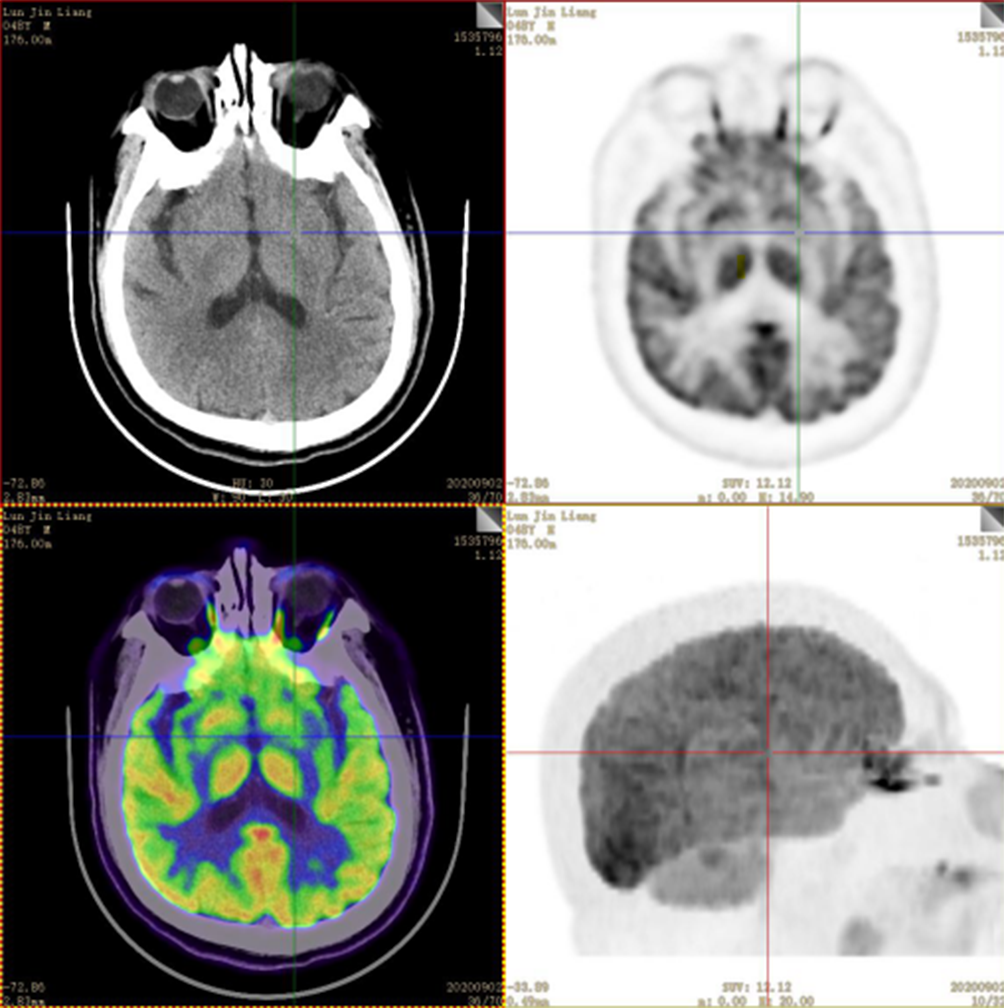

Supplement: Supplementary file 3 [file Image_1.TIF]
